# Global 30 meters spatiotemporal 3D urban expansion dataset from 1990 to 2010

**DOI:** 10.1038/s41597-023-02240-w

**Published:** 2023-05-26

**Authors:** Tingting He, Kechao Wang, Wu Xiao, Suchen Xu, Mengmeng Li, Runjia Yang, Wenze Yue

**Affiliations:** 1 Department of Land Management, Zhejiang University, Hangzhou, 310058 China; 2 Swiss Federal Research Institute WSL, Zürcherstrasse 111, CH-8903 Birmensdorf, Switzerland

**Keywords:** Developing world, Sustainability, Information technology

## Abstract

Understanding the spatiotemporal dynamics of global 3D urban expansion over time is becoming increasingly crucial for achieving long-term development goals. In this study, we generated a global dataset of annual urban 3D expansion (1990–2010) using World Settlement Footprint 2015 data, GAIA data, and ALOS AW3D30 data with a three-step technical framework: (1) extracting the global constructed land to generate the research area, (2) neighborhood analysis to calculate the original normalized DSM and slope height of each pixel in the study area, and (3) slope correction for areas with a slope greater than 10° to improve the accuracy of estimated building heights. The cross-validation results indicate that our dataset is reliable in the United States(R^2^ = 0.821), Europe(R^2^ = 0.863), China(R^2^ = 0.796), and across the world(R^2^ = 0.811). As we know, this is the first 30-meter 3D urban expansion dataset across the globe, which can give unique information to understand and address the implications of urbanization on food security, biodiversity, climate change, and public well-being and health.

## Background & Summary

As the wave of industrialization sweeps the world, the proportion of the global urban population continues to rise, reaching 56% of the total population in 2021^1^, and is expected to grow to 68% by 2050^2^. Urban area has been expanding for nearly a century, and it is projected that in the next 50 years, urban area in low-income countries, lower-middle-income countries, and high-income countries will increase by 141%, 44%, and 34%, respectively, compared to 2020. On the one hand, urbanization brings a lot of employment opportunities and better public services to society, on the other hand, it also led to global problems such as climate change^3,4^ and resource depletion^5–7^. Urban form is an important factor in describing the process and situation of urbanization, which affects the socio-economic conditions of urban areas^8^, urban climate^9^, public health^10^, energy consumption^11^ and other natural and social conditions to a large extent, while the study of urban three-dimensional spatial structure and its expansion process can provide a unique perspective for evaluating urban environment and studying human activities. The development of Earth Observation (EO) technology has made it possible to obtain large-scale global information, and various studies to measure urban form have emerged^12^. Many studies mainly focus on the urban horizontal form represented by the impervious surface range, depicting urban’s horizontal boundaries and evolution from different scales^13–16^. For example, Marcocini^17^ presented the 10-meter World Settlements Footprint in 2015(WSF 2015), Li *et al*.^15^ drew global city boundaries by acquiring the global impervious surface range time series dataset from 1985 to 2018. These studies captured the extent and boundaries of urban areas in the horizontal direction, which are critical for assessing sustainability challenges such as the urban environment, food security, biodiversity, etc.^18,19^. However, relying only on two-dimensional plane data cannot reflect the actual building density and land use intensity of urban areas^20,21^ and also ignores the heterogeneity of urban internal structure^22^. To measure urban morphology more accurately, it is essential to evaluate urban height and three-dimensional spatial structure.

So far, a few studies have focused on the vertical structure of cities, either on a small scale^23^ or on a global scale^24,25^. In terms of data sources, many researchers use high-resolution optical remote sensing images^26–29^, synthetic aperture radar (SAR) images^30–35^, and other data to extract building heights. For example, LIDAR^29^ can directly construct building height and morphology by acquiring 3D point clouds, and airborne radar^33^ can obtain multi-directional observation data of target buildings by virtue of stereo image pair inversion method, from which high-rise can be estimated. However, these methods are only applicable to the inscription of 3D structures in a small area and cannot be extended to the whole world with low cost and high accuracy. To address this gap, many institutions and companies have attempted to produce global-scale 3D urban datasets, such as OpenStreetMap 3D^36^, Google Earth 3D^37^, CityGML^38^, Microsoft Building Footprints^39^, etc. OpenStreetMap 3D^36^ is a dataset contributed by volunteers on the OpenStreetMap platform. This dataset contains a large amount of 3D building information for cities that can be downloaded or accessed using the API as needed. However, the quality of the dataset varies, with incomplete or inaccurate data for some areas, and the relatively low resolution of the dataset makes it unsuitable for high-precision applications. Google Earth 3D^37^ is a dataset created and maintained by Google Inc. that uses remote sensing technology and other data sources (such as aerial photography) to capture three-dimensional structural information of cities. The dataset has global coverage and high quality, and can be used for some high precision applications^40^. However, the dataset is not fully open for modification or update, and accessing the data requires costs and certain technology. CityGML is an international standard city model format that can be used to represent 3D structural information of cities, including buildings, roads, parks, etc. CityGML^38^ dataset is highly accurate and scalable, and can be created and edited by city planning agencies, municipalities or private companies. However, the dataset requires specialized software and technology to process and use, and there is a cost to acquire and update the datasets. Microsoft Building Footprints^39^ is a dataset created and maintained by Microsoft Corporation using remote sensing technology and deep learning algorithms. Microsoft uses satellite imagery, aerial photography and other data sources to capture building outline and height information for cities, and then uses machine learning algorithms to classify and analyze this information. The dataset contains contour data and height information for millions of buildings around the world and is of high quality, making it suitable for some high-precision applications. However, the coverage of the dataset is relatively small, containing data for only a part of the city, and the dataset is not completely open and cannot be modified or updated. In conclusion, most of these existing 3D urban datasets are not freely available due to their commercial properties, and either have restricted coverage or insufficient accuracy.

With the publication of open, globally freely available data, several studies of urban building height and 3D structure estimation at the global scale have been carried out by scholars around the world. More and more people are using machine learning methods to establish the relationship between building height and a series of multivariate data. Li *et al*.^41,42^ applied a random forest model based on mixed data to map continental-scale and global-scale 3D building structures with a resolution of 1 square kilometer. Frantz *et al*.^43^ used a machine learning regression model to extract building heights by combining Sentinel-1 and Sentinel-2 time series to draw a 10-meter spatial resolution building height map across Germany. Yang and Zhao^44^ created a dataset of building heights with a resolution of 1 km in China in 2017 using Spatial Information Gaussian Process Regression (Si-GPR) by incorporating spatial explicit/implicit information into a machine learning model. However, the quality of many machine learning models relies on the accuracy of reference data, and due to the inability to obtain high-quality cadastral data and open web map data in many countries and regions^44^, such models have limitations.

In summary, although the above studies have greatly enriched the research on urban height estimation, the previous studies mainly focused on the coarse-resolution building height estimation, while the globally high-resolution building height estimation still needs to be studied. In addition, most of the current research results of urban 3D structure are data at a specific time node, without time series information, and cannot reflect the spatiotemporal process of urban 3D expansion, which includes the improvement of infrastructure configuration in horizontal and vertical directions^45^. Most importantly, the commercial remote sensing software used in existing studies is not free. How to produce global building height products based on free open-source satellite data at low cost quickly and automatically is the main concern of our research. So far, only ALOS AW3D30 data is publicly available for the global height data, which is only available up to 2010^46^. Therefore, to supplement the current lack of high-precision, large-scale and long-term 3D urban expansion datasets, this study used World Settlement Footprint 2015 data^17^, GAIA data^47^, ALOS AW3D30 data^27,28^ to create the world’s first 30 m resolution 3D urban expansion dataset. The estimated city height data in 2010 are compared and verified with existing products.

## Methods

### Data collection

To supplement the current lack of high-precision, large-scale and long-term 3D urban expansion datasets, this study used World Settlement Footprint 2015 data^17,48^, GAIA data^47^, ALOS AW3D30 data^27,28^ and other ancillary data to create the world’s first 30 m resolution 3D urban expansion dataset.

The WSF2015^17,48^ is the global human settlements map at 10 m resolution for the year 2015^17^ and was generated through an advanced classification system that uses a combination of multi-temporal Sentinel-1 Synthetic Aperture Radar and Landsat-8 optical satellite imagery for the first time^49^. It is verified that WSF2015 significantly improves the detection of small rural settlements and better outlines scattered suburban areas. This dataset can be used for observations of any scale to support applications that require accurate and detailed information about human settlements and can be combined with other datasets to improve the accuracy of the analysis. In this study, the WSF2015 data was used to overlay (take intersect) with GAIA data to improve the spatial resolution of the study area (Table 2). Due to the irreversibility of urban expansion, i.e., the construction area in 2015 must contain the construction area in 2010, this study used the GAIA data in 2010 to clip the WSF2015 data to obtain the 10 m resolution construction area in 2010.

The GAIA data is a multi-temporal 30 m resolution global artificial impervious dataset from 1985 to 2018, containing annual change information^47^. It was produced with Landsat images, Sentinel-2 data, and night-time data. On the one hand, the GAIA data are used to extract the built-up area in 2010, which has the same time attribute as the AW3D30 DSM data^46^, and on the other hand, the GAIA data provide the time-series data of the construction area expansion(Table 1).

**Table 1:** Datasets used in this research.

Data	Type	Resolution	Time	Source
ALOS AW3D30 data	raster	30 m	2006–2010	ALOS@EORC Homepage (jaxa.jp)
GAIA data	raster	30 m	1985–2018	Finer Resolution Observation and Monitoring - Global Land Cover - 2015 v0.1 (tsinghua.edu.cn)
WSF2015	raster	10 m	2015	https://urban-tep.eu/puma/tool/?id=574795484&lang=en
JRC Global Surface Data	raster	30 m	1984–2022	https://global-surface-water.appspot.com/
Global Forest Change Data	raster	30 m	2000–2021	http://earthenginepartners.appspot.com/science-2013-global-forest
NASA DEM	raster	30 m	/	https://earthdata.nasa.gov/earth-observation-data
GRIP data	vector	/	2000–2015	https://sedac.ciesin.columbia.edu/data/set/groads-global-roads-open-access-v1/data-download
Building height reference data in China	vector	/	2010	https://amap.com/
Building height reference data in Europe	raster	10 m	2012	https://land.copernicus.eu
Building height reference data in the US	vector	/	2015	Publicly available data from the websites of local governments (see Table 2 for details).

The ALOS AW3D30 data are a global DSM with a 30 m horizontal resolution, and it is called “ALOS World 3D 30 m mesh” (AW3D30)^27,28^. It was produced based on a global 5 m grid version of the DSM dataset. As previous research showed^50^, it has a height accuracy of 4.38 m (standard deviation, STDEV) and 4.40 m (RMSE)^28^, better than the Global Digital Elevation Model(GDEM) and the Shuttle Rader Topography Mission(SRTM) data. In particular, it is has high accuracy in flat areas, similar to Tandem-X digital elevation model (DEM) data^46,51^. Hence, this study used AW3D30 DSM version 2 to generate building height estimation (Table 1).

To improve the accuracy of the extracted study area, JRC Yearly Water Classification History^52^ and Hansen Global Forest Change^53^, which contain annual change information at the spatial resolution of 30 meters(Table 1), were used to mask the GAIA data, remove surface areas that are not part of the built-up area, including vegetation cover areas and surface water body areas. In addition, the NASA DEM data (Table 1), which is a global digital elevation model produced by NASA, was used to calculate the slope of the ground^46^, and the GRIP dataset^54^ (Table 1), which is a global road vector dataset, was used to remove the road pixels from the estimated results^46^.

To validate our estimated dataset, we collected publicly available reference data from multiple sources worldwide. Specifically, building height reference data in Europe for the year 2012 were taken from Copernicus Global Land Service (https://land.copernicus.eu)(Table 1), which demonstrate gridded building height for 25 cities. For the US, we collected publicly available datasets from the websites of local governments for the year 2015 (https://hub.arcgis.com, see Table 2 for details) (Table 1). These datasets contain vector data of building footprints with vertical features for 27 urban areas, ranging from megacities like New York and Los Angeles to counties that only include small towns in distant places. For China, building height reference data for the year 2010, expressed as floor number, were available for 24 selected large cities (https://amap.com/) (Table 1). Here, we assumed that each level is 3 meters high for all building heights expressed as floor numbers^55^.

**Table 2:** Collection of 3D building dataset for the US used for model training and validation.

US City	Source
Southeast Michigan	https://maps.semcog.org/BuildingFootprints
Chicago	https://data.cityofchicago.org/Buildings/Building-Footprints-current-/hz9b-7nh8
LA	https://egis3.lacounty.gov/dataportal/2016/11/03/countywide-building-outlines-2014-update-public-domain-release/
Boulder	https://hub.arcgis.com/datasets/0d43652d038a4a0dbca68f0501151bb0_0
Fort Collins	http://hub.arcgis.com/datasets/7e577a14c83f4d83a6b58657c48027da_0/data?geometry=-105.502%2C40.489%2C-104.343%2C40.671
Norman	http://hub.arcgis.com/datasets/d68b0defa057465db7167d9260c90ad9_0
Austin	https://data.austintexas.gov/browse?q=footprint&sortBy=relevance
Albuquerque	http://datacabq.opendata.arcgis.com/datasets/e65e375b680345e0b21fa7585d83ce9c_0?uiTab=table
New York	https://data.cityofnewyork.us/Housing-Development/Building-Footprints/nqwf-w8eh
San Francisco	https://data.sfgov.org/Housing-and-Buildings/Building-Footprints-File-Geodatabase-Format-/asx6-3trm
Miami	https://mdc.maps.arcgis.com/home/item.html?id=ab4d3a61e60c441bbfc1098d701fc991
Sarasota	http://hub.arcgis.com/datasets/6c679d2949544274aee3bee8182c5611_0?geometry=-84.608%2C29.432%2C-78.802%2C30.265&page=9
Boston	http://boston.maps.arcgis.com/home/item.html?id=c423eda7a64b49c98a9ebdf5a6b7e135
Santa_Clara	http://hub.arcgis.com/datasets/ee83a3518a7249fda22866117463de3f_0?page=10
Reedsburg	http://hub.arcgis.com/datasets/dbe64a71897e4982934dbd7637d576d5_0?geometry=-91.204%2C43.336%2C-87.378%2C44.031
Macomb	http://hub.arcgis.com/datasets/b5bbaf4fef6e4b59b8214ccaa17b8331_0
Washtenaw	http://hub.arcgis.com/datasets/beefad4ecf334b43b883123a72bf86b7_0
Henderson	http://hub.arcgis.com/datasets/23e5f3506f034c3d99b84e54fce51584_11
Lincoln	http://hub.arcgis.com/datasets/1b6a5a2ef1b34c28950c4e720e8d7a3d_0
Monroe	http://hub.arcgis.com/datasets/61e3bf9f7da143b3b87e17dabe5b0c52_0
St._Clair	http://hub.arcgis.com/datasets/d00d7cffefd6466086dcfc7202f185f4_0/data?orderBy=MEDIAN_HGT&page=109
Sioux Falls	https://hub.arcgis.com/datasets/065e40f79b784848b403130234d95a1e_5?geometry=-97.326%2C43.445%2C-96.019%2C43.619&page=8145
Bernalillo	https://hub.arcgis.com/datasets/e65e375b680345e0b21fa7585d83ce9c_0
Arlington	https://hub.arcgis.com/datasets/bac045c94c144838a7e65fbcf7aa939c_0?page=1532
DC	http://opendata.dc.gov/datasets?q=3d&sort_by=relevance
Cambridge	https://www.cambridgema.gov/GIS/gisdatadictionary/Basemap/BASEMAP_Buildings.aspx

### Technical framework

**Fig. 1:**
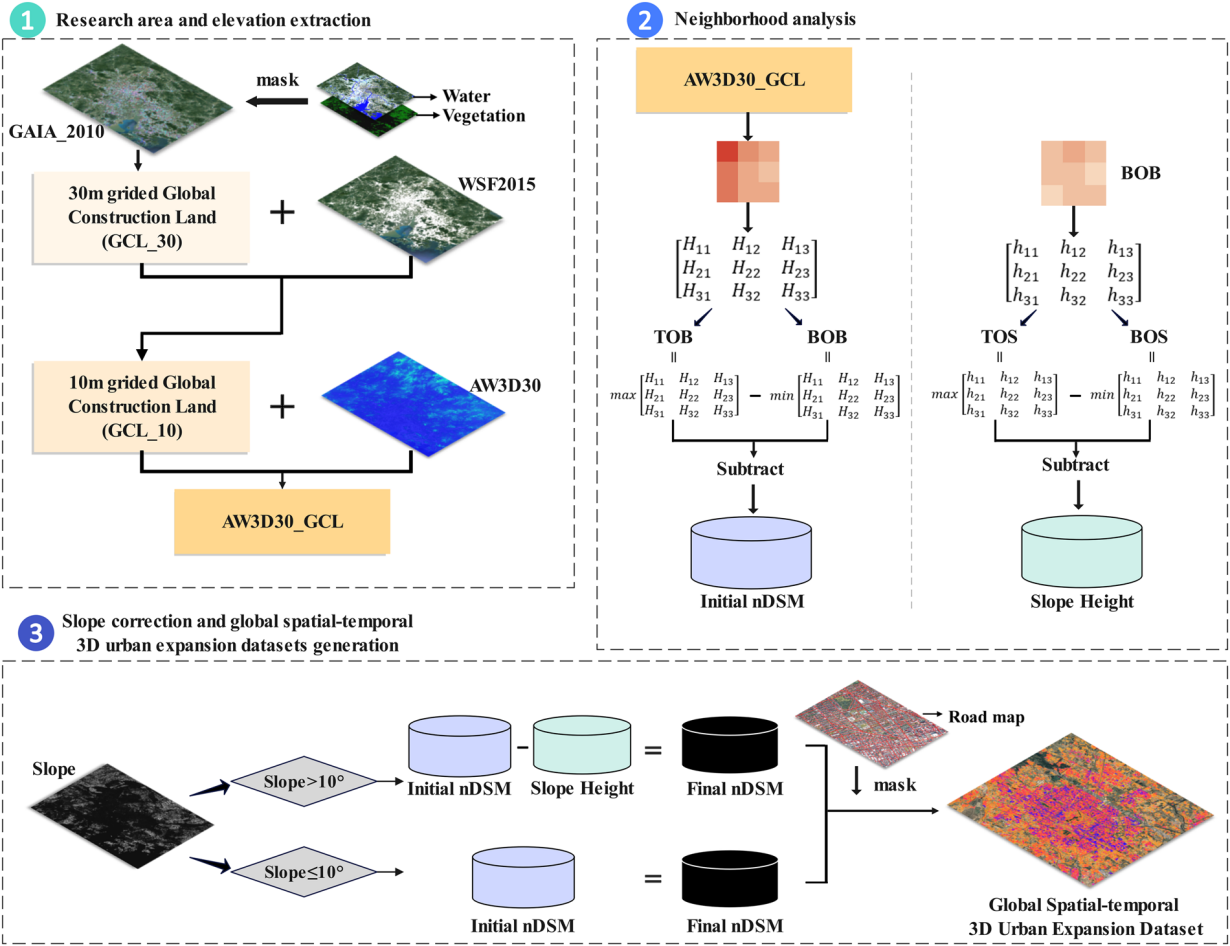
The technical framework of the research.

As shown in Fig. 1, the workflow for obtaining accurate global spatiotemporal 3D urban expansion datasets mainly includes three parts. The first part aims to extract the study area by overlaying GAIA and WSF2015 data. The AW3D30 data were then masked out with the study area to gain its elevation. The second part is mainly about the neighborhood analysis to obtain the initial nDSM and slope height in steep areas. The third part is to correct the slope error in areas greater than 10° and attain the final nDSM. The GRIP data were used to remove the road pixels in the resulting map^46^. Finally, the global building height data with a resolution of 30 m in 2010 and the spatiotemporal 3D urban expansion datasets are shown. The method is depicted in the flowchart (Fig. 1), and details are given in the following sections.

### Research area and elevation extraction

Since the ALOS AW3D30 data are global DSM data, we first needed to extract the global construction land as the research area of this study to mask the AW3D30 data. Global construction land refers to the land used for carrying infrastructure construction after removing water and vegetation from impervious surface coverage worldwide. GAIA is a global impervious surface extent time-series dataset containing data for each year from 1985 to 2018, to ensure that the data used in the study have consistent temporal properties, we obtained GAIA data for 2010 (GAIA_2010) as the preliminary extent of the study area by calculation with a spatial resolution of 30 m. Next, to further improve the accuracy of the extracted study area, we used JRC Global Surface Data^52^ and Global Forest Change Data^53^ to mask the water body pixels and green space pixels in GAIA_2010, and got Global Construction Land with 30 m resolution (GCL_30) and inherited the global impervious surface time-series expansion data starting in 1985, with a time resolution of 1 year. Then, by taking the intersection of the obtained GCL_30 data and the WSF2015 layer, we not only obtained a finer 10 m gridded Global Construction Land (GCL_10), which is the research area of this study, but also verified and improved the accuracy of the extracted research area through the World Settlement Footprint data. Thereafter, we overlaid the AW3D30 height data with GCL_10, i.e., added height information to each pixel in the GCL_10, to obtain the original global construction land surface elevation data in 2010 (AW3D30_GCL) and the original dataset of time-series 3D expansion of global construction land from 1990 to 2010.

### Neighborhood analysis

**Fig. 2:**
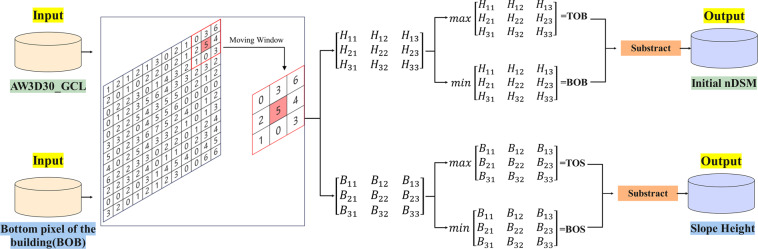
Schematic diagram of neighborhood analysis.

Since the AW3D30_GCL data represent the surface elevation rather than the height of the building, we performed neighborhood analysis on it and used moving window method to calculate the initial normalized DSM (nDSM) and slope height (SH) of each pixel in the study area (Fig. 2), which was practiced in China by Huang *et al*.^46^. Specifically, to obtain the initial nDSM, we first performed neighborhood analysis on each pixel in AW3D30_GCL, centering on the target pixel (the red pixel in Fig. 2), and establishing a moving window with a radius of 30 m. The 9 pixels contained in the moving window formed a weight matrix. The maximum value of the matrix element was defined as the top pixel of the building (TOB). The minimum value of the matrix element was defined as the bottom pixel of the building (BOB), and the height difference between the two was the original nDSM of the target pixel. On this basis, to correct the error caused by the slope of the ground, we calculated BOB’s slope height (SH). Similar to the previous operation, the neighborhood analysis method is applied to each pixel in the BOB to obtain the maximum and minimum values in a moving window centered on the target pixel, which was defined as the top pixel of the slope (TOS) and the bottom pixel of the slope (BOS), respectively, and the value subtracted from the two is the slope height (SH) of the target pixel.

### Slope correction and global spatiotemporal 3D urban expansion datasets generation

Huang *et al*.^46^ conducted a slope sensitivity analysis in China, demonstrating that when the slope of the terrain exceeds 10°, the average deviation and root mean square error between the estimated building height and the reference building height suddenly increase. Besides, we supplemented our analysis with a quantitative comparison of global and Chinese slopes to justify the choice of thresholds (SI Fig. 1). Results showed that the span of slope in China essentially covers slope span globally (the area of the global slope exceeding the maximum slope in China is only 33 km^2^), demonstrating that the thresholds generated in China are globally extensible. Hence, we carry out slope correction for areas with a slope greater than 10°. Specifically, we use NASADEM data (Table 1) to calculate the topographic slope of global construction land areas. The original nDSM calculated by neighborhood analysis was used as the final nDSM for pixels whose slope was less than 10°. While for areas with a slope greater than 10°, slope correction was performed, and the final nDSM was further calculated by subtracting slope height (SH) from original nDSM (Fig. 1). After obtaining the final nDSM of the global construction land, we used GRIP data^54^ to remove the road pixels in the result to further improve the accuracy of building area identification and increase the readability of the resulting map. Finally, the global 30 m resolution building height data in 2010 and the global urban 3D spatiotemporal expansion dataset were obtained.

## Data Records

**Fig. 3:**
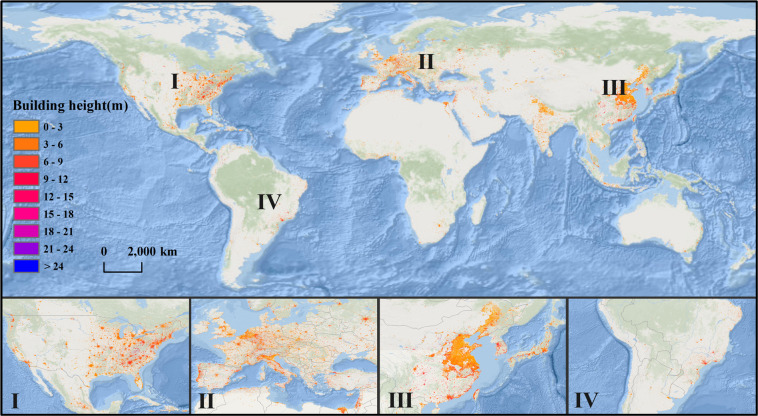
Global 30 m resolution building height distribution in 2010.

Data described in this paper can be accessed at 10.6084/m9.figshare.21792209.v2^56^. This dataset contains 621 GeoTiff files of 5° grids, each file contains building height information at 30 m resolution and information of the year the building was built from 1990 to 2010, which combined can reflect the urban 3D expansion phenomenon. This dataset contains annual urban 3D structure in 20 years, and can be visualized and processed by GIS software. For example, Fig. 3 shows global 30 m resolution building height distribution in 2010.

## Technical Validation

### Accuracy validation of global height data

**Fig. 4:**
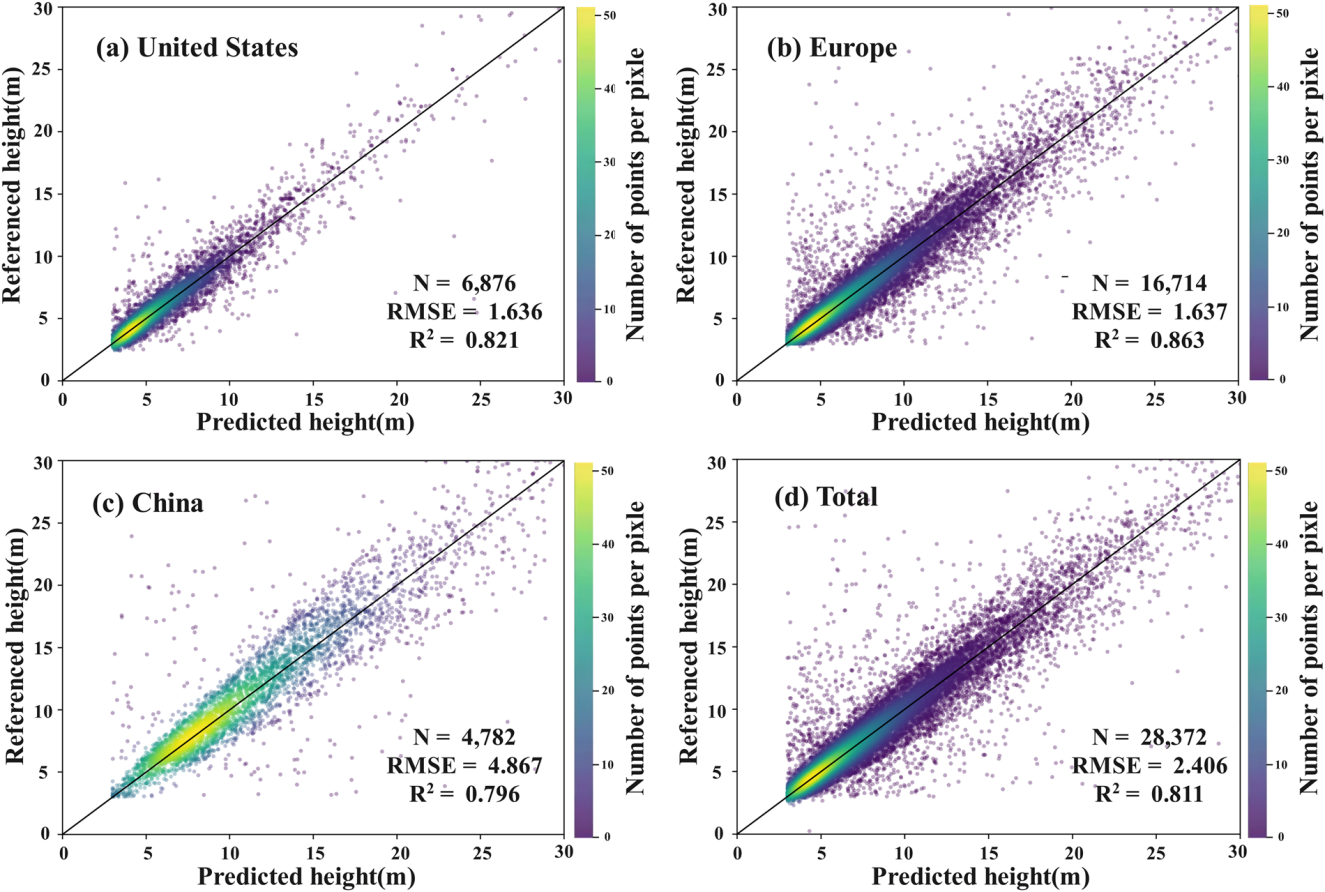
Validation of the estimated building height with reference building height data in three regions. (**a**) United States; (**b**) Europe; (**c**)China.

We collected building height reference data from multiple sources for the United States, Europe and China, and compared them with our estimated height values (Fig. 4). Most of the evaluation points are distributed near the 1: 1 line, showing that the predicted building height in each region are generally consistent with reference height officially released worldwide. Specifically, building height from this study and the reference height data in the United States are largely scattered along the 1: 1 line, presenting low values of the Root Mean Square Error (RMSE) smaller than 1.65 (Fig. 4). Independent testing indicates that R^2^ values of the dataset for the United States, Europe, China equal 0.821, 0.863, and 0.796, respectively, while R^2^ value for all region equals 0.811(Fig. 4). Additionally, Fig. 4 shows that the average building height in China exceeds that of other regions, at about 8 m, compared to about 5 m in other regions.

### Comparison with global building height products

**Fig. 5:**
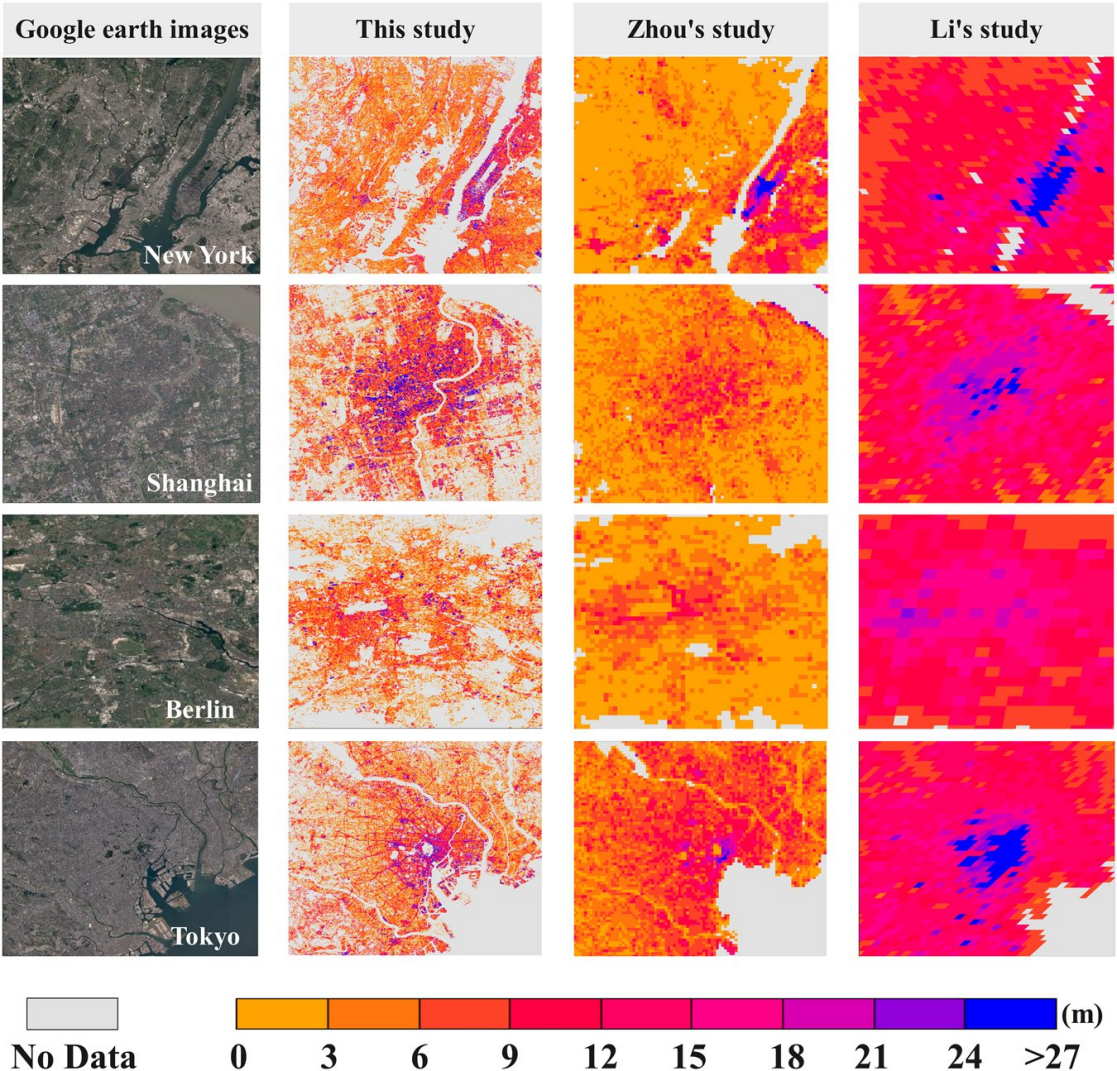
Comparison of estimated building height maps by our method and methods of Zhou *et al*. (2022) and Li *et al*. (2022) in New York, Shanghai, Berlin, and Tokyo.

In order to compare our global urban 3D structure more intuitively, we compared it with the product data obtained by several similar researches. Figure 5 compares the building height maps estimated in this study with the results of Zhou and Li, while SI Fig. 2 in Supplementary Information compares the building height maps estimated in this study with the results of Huang. Overall, our building height map and the other two findings show a consistent spatial distribution trend of decreasing building heights from the city center to the suburbs. However, our dataset has an extremely high spatial resolution of 30 m, compared with Zhou’s 500 m and Li’s 1000 m. The fine resolution allows our maps to accurately identify small spreads of non-building areas such as water bodies and green spaces in the city and exclude them from the results. Therefore, compared with Zhou’s results, our results more accurately identify and mask non-built-up areas in the suburbs, without the saturation effect of large tracts of low-rise built-up land. In addition, Li’s height estimation results show an overestimation of building height in the city center^46^. One of the reasons for this problem is that the excessively coarse spatial resolution leads to errors in the height distribution of the city center. At the same time, Li’s results overestimate the building heights in suburban areas, mainly for low-rise buildings. In contrast, our model performs well in both downtown and suburban areas, possibly due to the height sensitivity of the AW3D30 DSM in detecting small and low-rise buildings^46,51^.

**Fig. 6:**
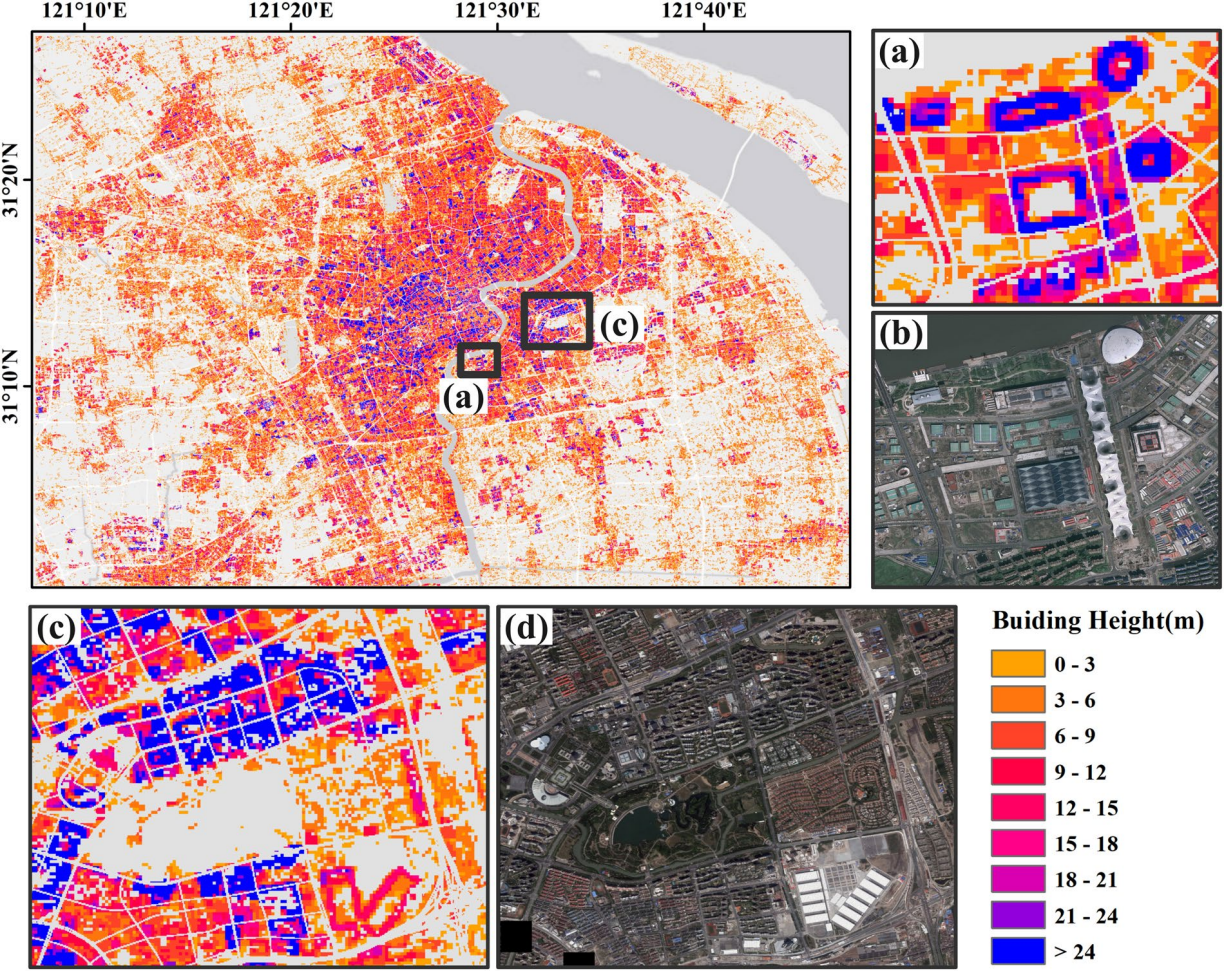
Building height close-ups in Shanghai at cell size of 30 m by 30 m. (**a,b**) Shanghai World Expo Park; (**c,d**) Shanghai Science and Technology Museum and Shanghai New International Expo Center.

Compared with other existing urban 3D structure datasets, our dataset has a very fine granularity, which can simultaneously describe the outer contour and building height of the building. Figure 6 shows the detailed distribution of building heights in Shanghai. The residential pattern in Shanghai shows a trend of being higher in the city center and gradually lower towards the surrounding areas, both at the horizontal and vertical levels. Our estimation results show that Shanghai’s old city centered on the west bank of the Huangpu River has the densest and tallest building distribution. Among them, super high-rise buildings exceeding 24 m account for more than half of the same type of buildings in Shanghai. The farther away from the city center, the sparser the building density and the lower the average height, and the proportion of permeable surfaces such as water bodies and green spaces in land use types gradually increases. Figure 6a–d show the estimation results and the corresponding high-resolution imagery collected from Google Earth. The close-up of various venues in the Shanghai World Expo site in Fig. 6a shows that our estimation results can capture the details of the buildings well. Figure 6c not only shows the semi-circular Shanghai Science and Technology Museum and the double-rectangular Shanghai New International Expo Center, but also identifies the high-density high-rise residential area in the north and the low-density low-rise villa area in the east. Building height close-ups in Berlin, Tokyo and São Paulo are provided in Supplementary Information (SI Figs. 3–5).

## Usage Notes

### Urban applications

**Fig. 7:**
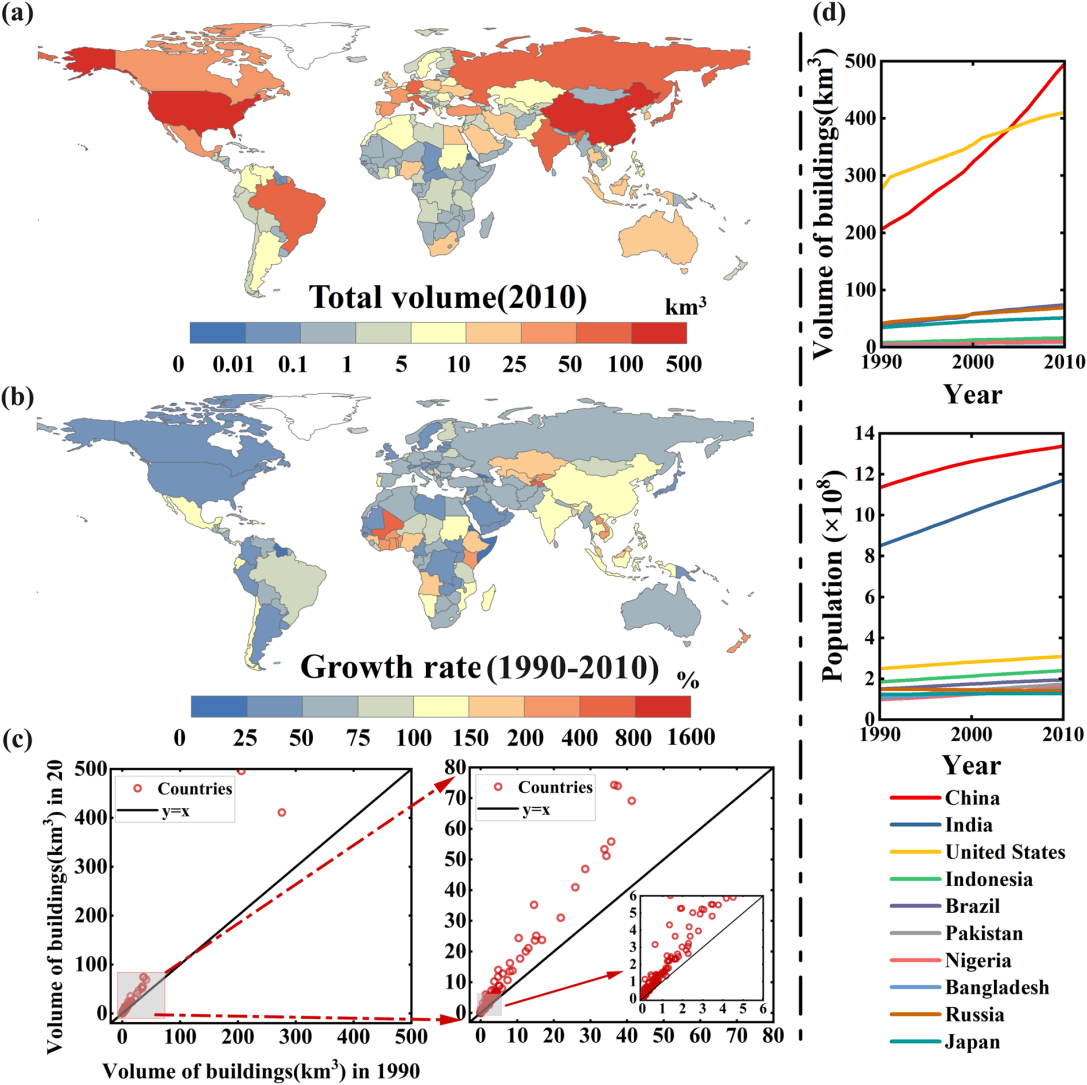
Dynamics and patterns of urban 3D expansion at the country level. (**a**) Total volume of national-scale buildings worldwide in 2010. (**b**) Total growth rate of global national-scale building volume from 1990 to 2010. (**c**) Comparison of global total building volume by country in 1990 and 2010. (**d**) Dynamics of building volume and population from 1990 to 2010 in the top 10 countries ranked by population in 2010.

To further illustrate the potential use of this dataset, we performed an example analysis of patterns and dynamics of global spatiotemporal 3D urban expansion at the country level, which could be used to further understand the temporal property of the dataset. The dynamics and patterns of urban 3D expansion in each country during 1990–2010 are shown in Fig. 7a,b. Figure 7c shows the comparison of the building volumes of various countries in 1990 and 2010. In densely populated countries, the dynamics and patterns of urban 3D expansion vary over time and space, and are not completely consistent with the dynamics and patterns of population growth over two decades (Fig. 7d). We selected the top 10 countries by population in 2010 (https://data.WorldBank.org, last accessed: December 2, 2022) to map. As the second most populous country in the world, India’s building volume is much smaller than that of China and the United States. In 2010, the per capita building volume is only 63.46 m³/person, far smaller than China’s 370.88 m³/person and the United States’ 1328.62 m³/person.

### Advantages and limitations

This study not only updates and refines the findings of Huang *et al*., but also extends them from a spatiotemporal perspective. Firstly, in the spatial perspective, we have refined the resolution of construction land globally to 30 m as never before, enabling a more accurate portrayal of the three-dimensional urban building form rather than just the approximate height trend. Secondly, from the perspective of time, we obtained the time-series change data of urban 3D expansion from 1990 to 2010, which can not only intuitively show the dynamic process of three-dimensional expansion of cities in decades, but also apply the time-series change algorithm to analyze the 3D shape of future global cities. However, it should be noted that since ALOS AW3D30 data only provide height data up to 2010, the endpoint of the time-series dataset obtained in this study is 2010. In addition, in the slope correction, the threshold of 10° was determined with reference to a similar study done in China by Huang *et al*. Although the threshold obtained in this region has general applicability on a global scale due to the complexity of the Chinese terrain (which almost covers the global extreme terrain slope), there is still room for improvement in the threshold selection for future studies. To fill this gap, in future studies, we will use the existing remote sensing dataset to invert the 2010 results to 2030 to perform future-oriented prediction research, and we will also focus on sub-regional validation by collecting real data and comparing the errors between real data and height data generated by neighborhood analysis to make the best threshold selection.

## Supplementary information

Supplementary Information-Global 30 meters spatiotemporal 3D urban expansion dataset from 1990 to 2010

## Data Availability

The programs used to generate the datasets and all the results were ESRI ArcGIS (10.8.1) and Google Earth Engine (GEE). The scripts of data collection and pre-processing on GEE can be accessed on GitHub (https://github.com/XinYijie/GlobalBuildingHeight).
