# Supplementary material for: Global 30 meters spatiotemporal 3D urban expansion dataset from 1990 to 2010
Source: Sci Data. 2023 May 26;10:321. doi: 10.1038/s41597-023-02240-w (PMC10220007; doi:10.1038/s41597-023-02240-w)
Supplement: Supplementary file 1 — Supplementary Information-Global 30 meters spatiotemporal 3D urban expansion dataset from 1990 to 2010 [file 41597_2023_2240_MOESM1_ESM.pdf]

Supplementary Information

Table of Contents:

SI Figure 1	Page 2
SI Figure 2	Page 3
SI Figure 3	Page 4
SI Figure 4	Page 5
SI Figure 5	Page 6

We compared the area under each slope globally and in China, and took the area logarithmically for better graphical presentation, as shown in SI Figure 1.

The slope span of China is 0-52° and the global slope span is 0-72°, where the area of the area with slope over 52° is 33km^2 in total, which is a small area proportion and produces negligible impact.

In addition, the area where the topographic slope exceeds 52° (exceeding the maximum slope in China) on a global scale is the Himalayas, which are located between the borders of Nepal and China. This region includes some of the highest peaks in the world (e.g., Mount Everest) and has unusually steep topography with relatively deep valleys and canyons between the peaks, creating extremely high topographic gradients. These regions are not suitable for urban construction sites due to their huge topographic undulations and harsh natural environment, and therefore have no impact on the slope correction of urban areas in this paper.

In summary, by quantitatively comparing the global and Chinese slope spans, it can be shown that the selected threshold (10°) for the slope correction under Chinese slope conditions can be extended to the global scale.

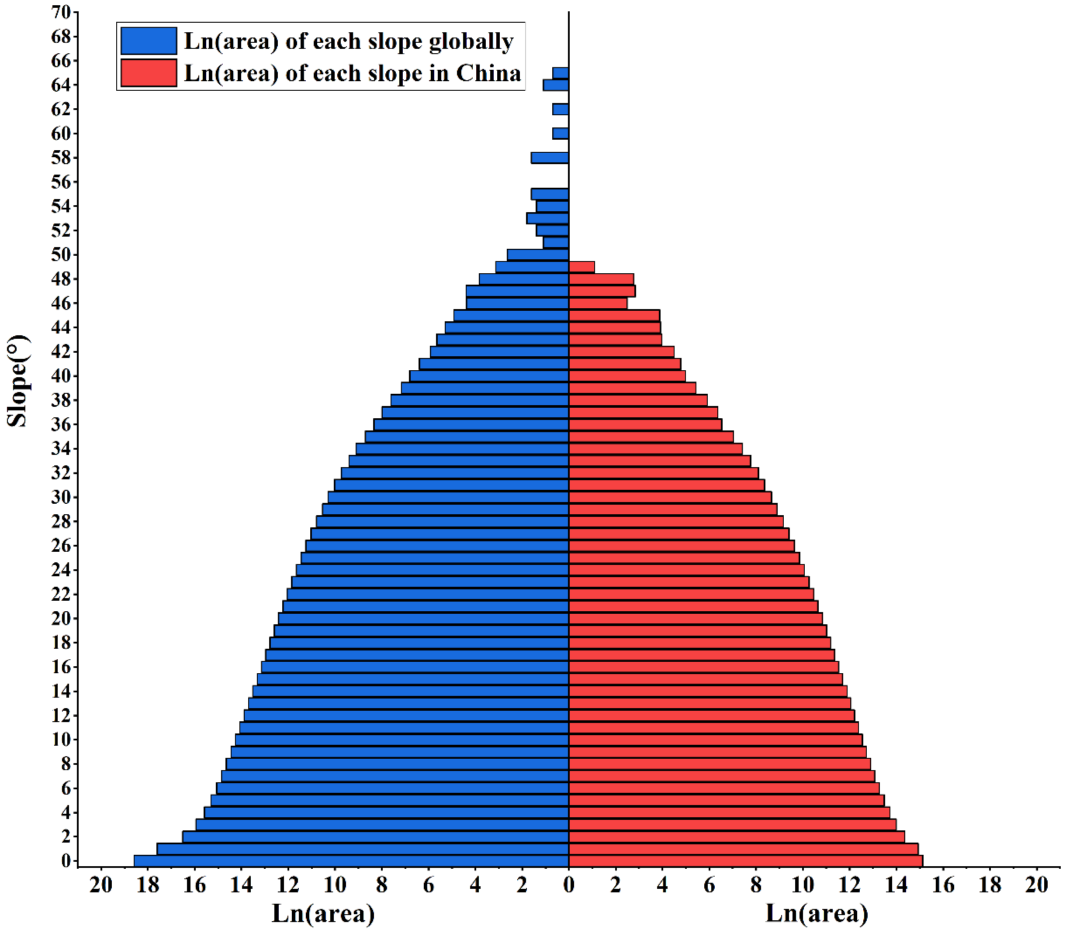

SI Figure 1 Comparison of the area corresponding to each slope globally and in China.

To compare our building height estimation results with those of Huang et al., we selected four cities: Beijing, Shanghai, Guangzhou, and Wuhan, which are shown in SI Figure 2.

Compared with Huang's results, our results have three improvements:

1. Our results have a higher spatial resolution (10m raster for ours compared to 30m raster for Huang's), thus can better distinguish between construction and non-construction land in the horizontal direction;
2. Our results exclude some of the overestimated built-up areas, avoiding the saturation effect of high values in the city center;
3. We extend our results to the world and not only to China.

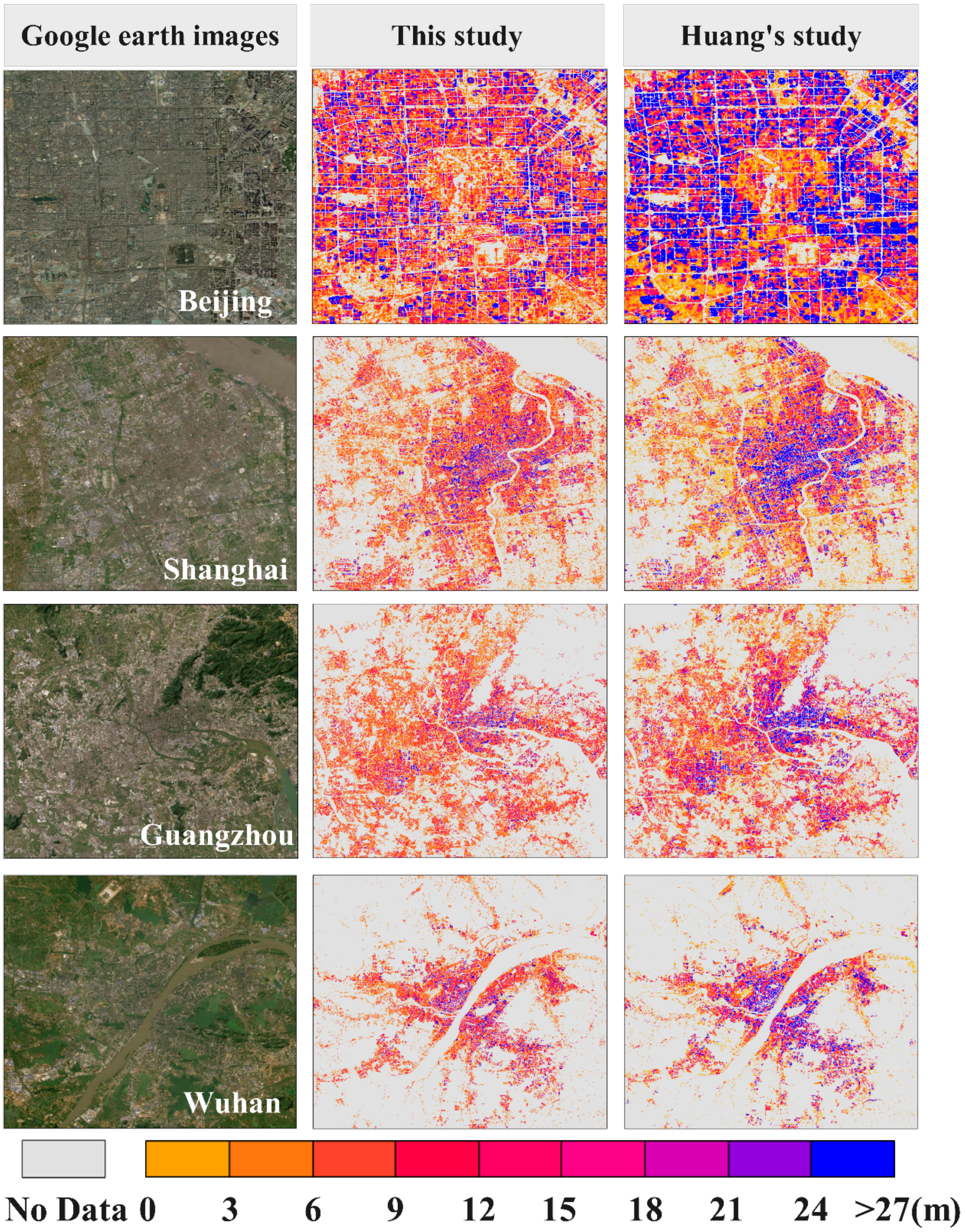

SI Figure 2 Comparison of estimated building height maps by our method and methods of Huang et al. (2022) in Beijing, Shanghai, Guangzhou, and Wuhan.

To better illustrate the expressiveness of the dataset generated in this study, we have added more detailed comparison images. The following three supplementary figures below show building height close-ups in Berlin, Tokyo and São Paulo, respectively.

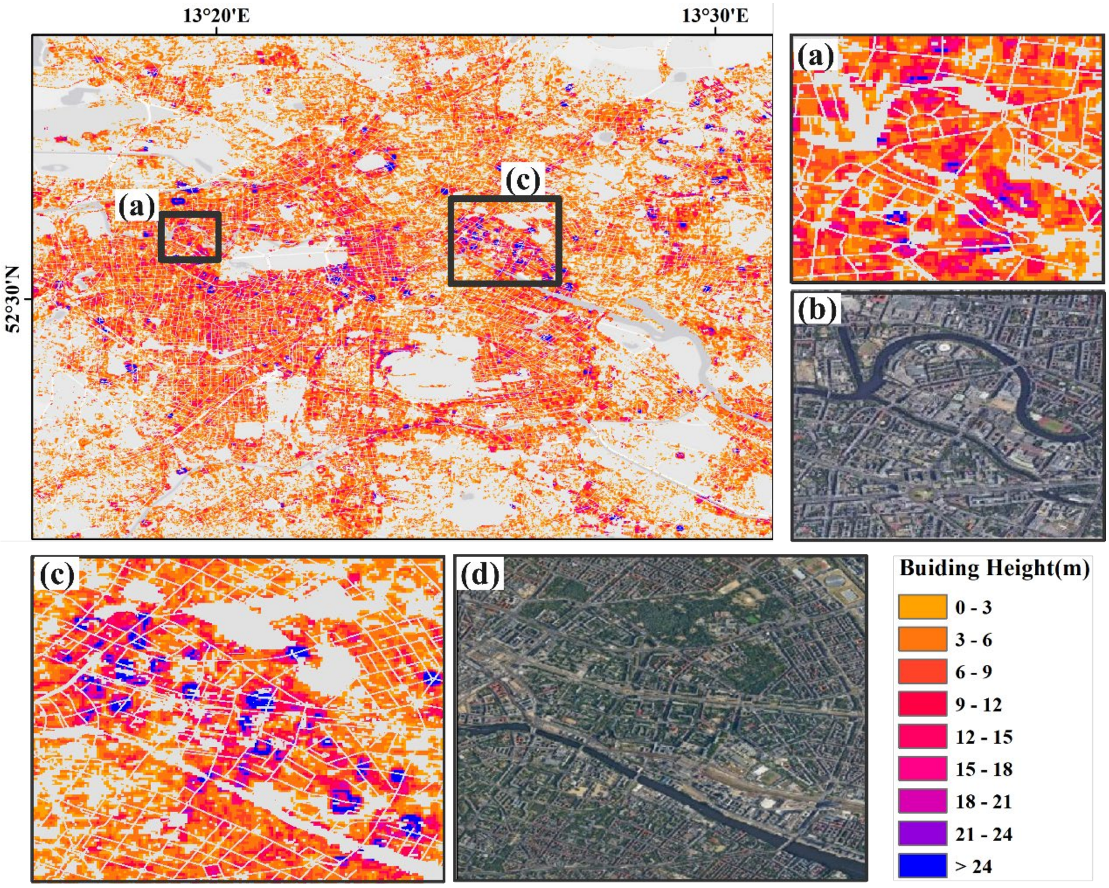

SI Figure 3 Building height close-ups in Berlin at cell size of 30 m by 30 m.
(a)(b) Spree River and its surroundings; (c)(d) Friedrichshain Park and its surroundings.

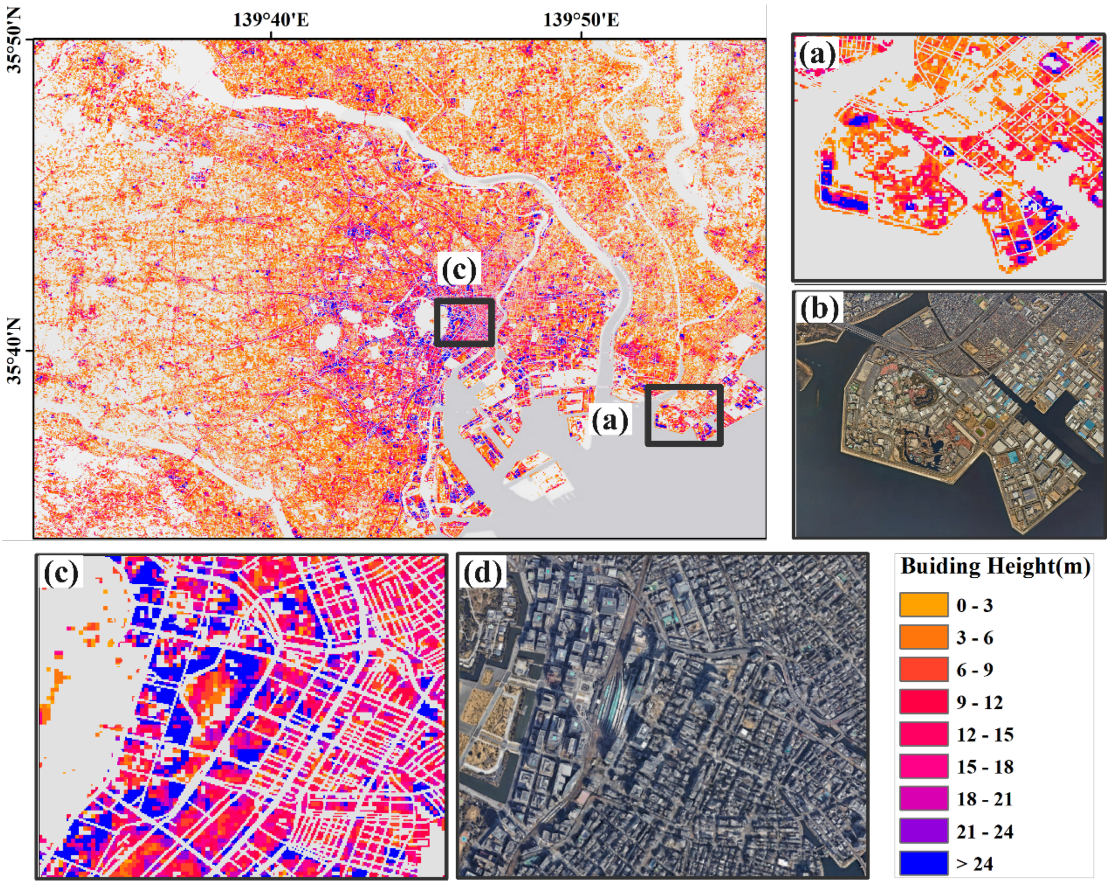

SI Figure 4 Building height close-ups in Tokyo at cell size of 30 m by 30 m. (a)(b) Tokyo Disneyland and Ocean Park; (c)(d) Daimaru Tokyo Mall and its surroundings.

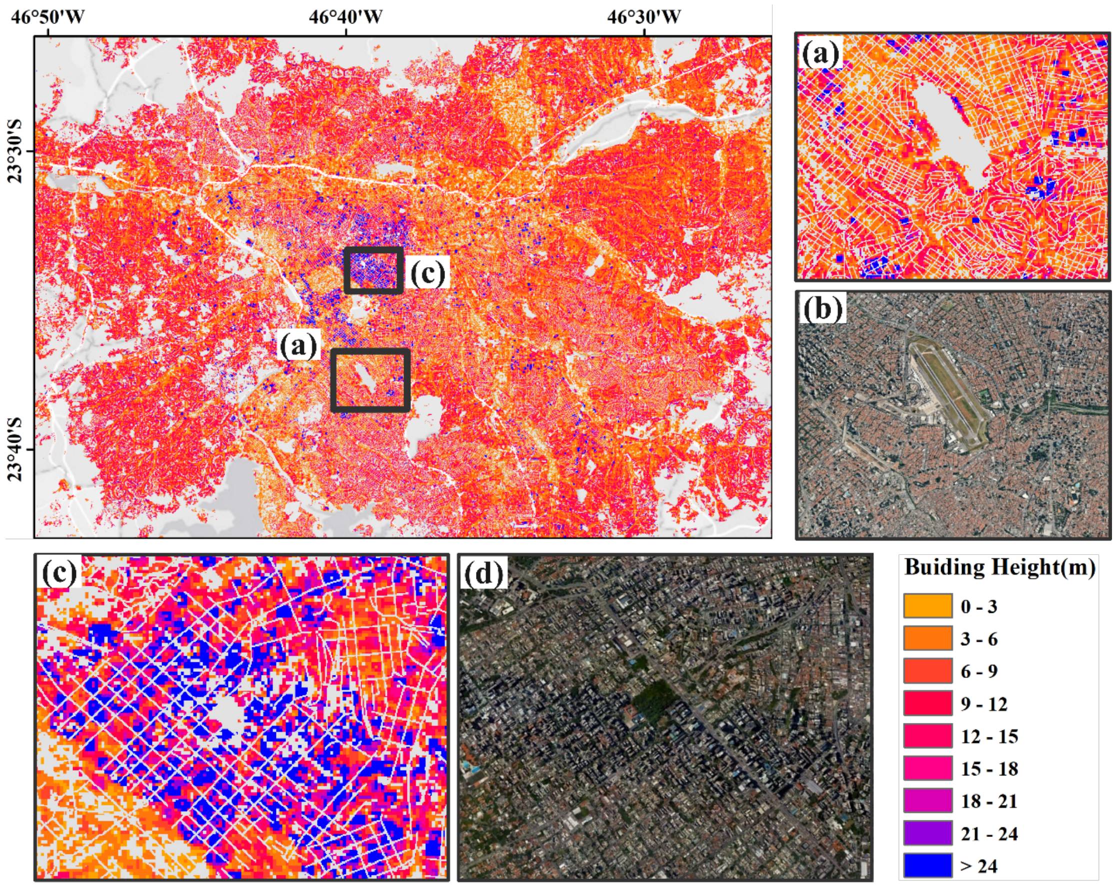

SI Figure 5 Building height close-ups in São Paulo at cell size of 30 m by 30 m. (a)(b) Congonhas Airport; (c)(d) Trianon Park and its surrounding downtown buildings.
